# Supplementary material for: Entomological surveillance of mosquitoes (Diptera: Culicidae), vectors of arboviruses, in an ecotourism park in Cachoeiras de Macacu, state of Rio de Janeiro-RJ, Brazil
Source: PLoS One. 2021 Dec 23;16(12):e0261244. doi: 10.1371/journal.pone.0261244 (PMC8699951; doi:10.1371/journal.pone.0261244)
Supplement: Attachment — Submitted filename: Response to Reviewers.pdf [file pone.0261244.s003.pdf]

Rio de Janeiro, July 2021

Dear Editor,

We are grateful for the considerations and suggestions for adjustments that greatly contributed to the enrichment of the paper. All suggestions made by the reviewers were rigorously considered and accepted.

Initially, we requested the inclusion of Universidade Iguaçu - UNIG in the affiliations of the last author of this article.

In relation to the included maps, we assure the editor of the total authorship of the images, being the main author of this article, Thamiris Balthazar. We also guarantee to the editor that the layers used to make these images are from an open and public database: Brazilian Portal for Open Data (https://dados.gov.br/dataset/malha-geometrica-dos-municipios-brasileiros) and the United States Geological Survey "LandsatLook" platform (http://landsatlook.usgs.gov /viewer.html).

Thus, we requested the alteration of image 1, where the authorship information was included in the caption, as well as changing the caption of this figure, also including a caption. We also requested the modification of images 2 and 3, which, together with image 1, went through the Preflight Analysis and Conversion Engine (PACE) digital diagnostic tool, as requested. Included in the legend of figures 2 and 3, the database used to prepare the image.

About the subtitles pointed out by the editor, we inform you that there was a typo in the order of the figures. Thus, the legend named figure 3 referred to figure 2, as well as the legend in figure 4 referred to figure 3. Therefore, there is no fourth figure to be inserted in this article.

In the corrected version of the manuscript, you will also be able to check the changes requested by the reviewers, all of which were accepted. Are they:

In line 37, the conclusion was changed, pointing to the importance of entomological surveillance in ecotourism parks.

On line 133, the word "sacrificed" has been replaced by the word "preserved".

In line 279, a continuation of the paragraph was included pointing to the articles that refute the vectorial capacity of *Culex quinquefasciatus* in Zika virus transmission.

Thanks again to the editors and reviewers.

Best regards,

Thamiris D’Almeida Balthazar

E-mail: thamirisbalthazar@gmail.com

Fone: +55 (21) 972291756 / (21) 25621232
